# Evidence for Sequential Performance Effects in Professional Darts

**DOI:** 10.3389/fpsyg.2018.00591

**Published:** 2018-04-26

**Authors:** John F. Stins, Gur Yaari, Kevin Wijmer, Joost F. Burger, Peter J. Beek

**Affiliations:** ^1^Department of Human Movement Sciences, Faculty of Behavioural and Movement Sciences, Vrije Universiteit Amsterdam, Amsterdam, Netherlands; ^2^Bioengineering Program, Faculty of Engineering, Bar-Ilan University, Ramat Gan, Israel

**Keywords:** hot hand, darts, sequential effects, independence, motor programming

## Abstract

**Objectives:** The study of sequential effects in aiming tasks might shed light on the organization of repetitive motor performances over time. To date, investigations of such effects in sports have been limited and yielded mixed results. Given the relatively short time intervals between successive attempts, and the absence of defensive interventions, dart throwing provides a potentially fruitful testing ground for examining the presence of sequential performance effects in the motor domain.

**Methods and Results:** A total of 80 competitive darts matches of 10 of the world’s best players were scored from publicly available video footage in terms of sequences of hits and misses of triple 20. In darts, throws are organized in legs, i.e., a rapid succession of three throws by the same player, allowing us to investigate various transitions in performance (throw 1 → 2, 2 → 3, and 3 → 1). The resulting binary sequences were analyzed statistically in terms of independence and stationarity. Across players significant statistical evidence was found for sequential dependence from the first throw in a leg to the second throw, but not for the other transitions. As regards to stationarity, a significant decline in performance was observed in the course of the match.

**Conclusions:** In professional darts, evidence can be found for both sequential dependence as well as for non-stationarity, implying that performance does not, or at least not always, constitute a stationary random independent process. More research is needed on the motor control mechanisms underlying the observed carry-over effects within triplets as well as the possible causes of non-stationarity.

## Introduction

Many sports involve aiming movements, whereby the athlete or player attempts to propel an object toward an intended target location. Examples include golf, darts, basketball, archery, bowling, and snooker. Such sports are typically characterized by high accuracy demands and hence accurate and consistent motor control. Even within professionals, however, performance is seldom constant and exhibits considerable variation over time, for example due to sensorimotor ‘noise’ or fluctuations in attention, arousal or fatigue over the course of a match or tournament. An unanswered question is whether variation in performance contains subtle regularities that are not visible to the naked eye, and could not be described as ‘white’ uncorrelated noise. In the current study we examined whether performance in successive dart throwing exhibits sequential dependencies and non-stationarities.

The search for regularities in performance sequences is motivated by the informal observation that players appear to exhibit streaks of very successful or very unsuccessful performances, deviating significantly from the overall mean skill level. That is, hits and misses appear to cluster in shorter or longer bouts, which seems highly unlikely from the assumption that successive attempts constitute a stationary random independent process (or more specifically, a Bernoulli process, considering the binary character of hits [0] and misses [1]). Given that extensive data sets in many different sports are nowadays readily available, this has led to considerable interest in the question whether performance follows such a Bernoulli process, or whether there is some kind of statistical dependence, in that performance is influenced by past events, and/or non-stationarity, in that the probability of hits and misses is not constant but varies over time. Statistical dependence implies that event sequences carry some form of ‘memory,’ while non-stationarity implies that another underlying factor, like fluctuations in arousal or alertness, influences the temporal structure of observed events.

A hotly debated phenomenon is the presence (illusory or real) of the so called ‘hot hand’ (see for example the analysis in The New York Times of October 17th, 2015) (Johnson, 2015). The hot hand can be described as the belief (held by players and spectators alike) or indeed the actual fact that “the performance of a player during a particular period is significantly better than could be expected on the basis of the player’s overall record” (Bar-Eli et al., 2006, p. 525; see also Gilovich et al., 1985). That is, the performance of the athlete temporarily increases well above his or her overall skill level. This is also sometimes called ‘being in the zone,’ or simply ‘being hot.’ In statistical terms, this phenomenon may occur because the outcome of a particular event, such as a basketball throw aimed at the hoop, is positively correlated with the outcome of the immediately preceding event^1^ (dependence), or because the probability of success is temporarily elevated (non-stationarity).

Two lines of research are pursued to address this topic. One line of research examines the subjective belief of players, fans and coaches regarding the occurrence of such spells of superior performance. A consistent finding is that humans tend to see evidence for hot hand performance, even when the underlying process is entirely random (given a certain performance level of the player). This misguided belief is due to the way our cognitive system processes information, and is also responsible for the so called gambler’s fallacy (e.g., Tversky and Kahneman, 1974). A second line of research (references below) examines sets of behavioral data, and tries to assess whether, and to what extent, deviations from randomness actually occur in performance. In the present study we follow this second line of research, and examine whether non-random sequences occur in professional dart throwing. To set the stage for this, we first describe previous studies that have examined this issue in different competitive fields.

Gilovich et al. (1985) were the first to examine the perceived and factual probability of success in sequences of basketball shots. They found that players and fans believed that the chances of hitting a shot were larger immediately following a successful shot (*hit–hit*) than following a miss (*miss–hit*). However, detailed analysis of professional basketball records obtained in 1980–1981 revealed “no evidence for a positive correlation between the outcomes of successive shots” (Gilovich et al., 1985; p. 295). In other words, when random sequences are presented, observers tend to fall prey to a powerful cognitive illusion whereby occasional positive serial correlations are given more weight than negative serial correlations.

Gilden and Wilson (1995) addressed the same issue in golf putting and darts. Analysis of the outcome sequences provided evidence for bouts of hits and bouts of misses to cluster together, such that performance exhibited a wave-like shape over time. However, in this study data were gathered from novice performers who still had to actively master the skills in question. As a result, it is unclear whether the results obtained also hold for experts, whose performance level is more stable and less amenable to short-term improvements due to practice.

Smith (2003) examined horse shoe pitching, using data of the 2000 and 2001 World Championship. In horse shoe pitching, players repeatedly toss a horse shoe toward a stake. When the horse shoe encircles the stake (a so called ringer), the pitch is considered successful, and points are awarded. Analysis of occurrences of ringers revealed modest evidence for hot hand spells.

Raab et al. (2012) asked whether the hot hand exists in volleyball, by analyzing sequences of hits (defined as a point scored by an attacker) and misses (errors such as fouls, or a ball smashed into the net). Analysis of the results of individual top players, using autocorrelations, revealed that streaks of hits and misses were not entirely random, reflecting hot hand-like behavior. However, the authors acknowledged that the results could well be more complicated because performance of individual players is likely also influenced by the behavior (successful or not) of the opposing team.

Bar-Eli et al. (2006) conducted a thorough review of hot hand research. One of their main findings was that evidence for the existence of hot hand was limited, but that considerable variation exists in the type of sports considered and the adopted statistical procedures. Importantly, the authors concluded that the emergence of a hot hand may in fact be strongly dependent on (as of yet unknown) psychological and emotional variables. A more recent meta-analysis, encompassing a variety of sports, concluded that the hot hand effect is probably non-existent (Avugos et al., 2013).

However, two recent studies shed new light on the hot hand phenomenon. They applied dedicated statistical techniques to examine sequential effects in free basketball throws (Yaari and Eisenmann, 2011) and bowling (Yaari and David, 2012), using data of professional tournaments (see also Bocskocsky et al., 2014). In both studies, the analyses revealed that the probability of success was not constant, and appeared to slightly increase or decrease over consecutive (successful or unsuccessful) attempts, which would be suggestive of hot hand trends. On the other hand, the authors concluded that it would be overly simplistic to claim that a given trial would be dependent on the outcome of the previous trial. That is, performance seemed to cluster in ‘good’ periods and ‘bad’ periods. The authors also concluded that the interpretation of these probability fluctuations was hampered by the absence of a clear psychological or physiological framework that could account for the observed patterns. Two other studies showed another type of deviation from the repeated independent Bernoulli trials hypothesis in basketball. In these studies (Neiman and Loewenstein, 2011; Attali, 2013) the authors analyzed the probability of taking a current shot given a success or failure in the previous shot, and the probability of succeeding given the result of the previous shot. These studies showed systematic deviations in both probabilities: the probability of the next shot being successful increases if the previous shot was successful, and the probability of succeeding in the next shot decreases if the previous shot was successful. These “anti-hot hand” effects could be explained by defense adaptation and the varying conditions between two consecutive shots; the defense adapts (thus reflecting strategic decisions) and makes the next shot harder to take (although this explanation arguably renders the term “anti-hot hand” less appropriate).

In the present study, we examined temporal fluctuations in the probability of success in darts. Dart throwing is similar in many respects to the sports activities described above, in that performance can be dichotomized in terms of success and failure, and in that players often generate large sequences of trials. However, a crucial difference pertains to the time interval between successive attempts, which is generally much shorter in darts than in any other game. Professional darts players throw triplets of darts with little time in between, whereas the time interval between triplets is comparatively much longer because players are taking turns during a match. From a classical motor control perspective (e.g., Schmidt, 1975), it could be argued that each throw involves the parameterization of a motor program, specifying motor variables such as joint angle, release speed, etc. Once a given throw is successful, the following throw should involve executing the same motor program (i.e., using the same parameter values), as this is still presumably stored in motor working memory. The longer the player delays the next throw, the more likely the memory trace will fade, which would require setting the motor parameters anew.^2^ In view of the relatively short time interval between successive attempts within triplets, in combination with the absence of defensive interventions, darts seems optimally suited for studying the presence of sequential performance effects.

We know of two studies that examined sequential effects in dart throwing: the study by Gilden and Wilson (1995) described above, and more recently the study of van Beers et al. (2013). Both studies found evidence of sequential dependencies. Crucially, the data were gathered in a lab setting, whereby players had to aim for a pre-specified region in the darts board. But we know of no study that examined sequential effects of elite players during live tournaments. To this end, in this exploratory study we looked for statistical patterns in sequences of dart throws using data of world ranking darts players. We adopted the dedicated statistical technique introduced by Yaari and Eisenmann (2011), Yaari and David (2012), in order to add to the evidence for or against sequential performance effects in sports. In addition, the study could provide insight into the strategies used by top darts players to accommodate fluctuations in motor output, and hence performance, which could be examined further in research on dart throwing from a motor control perspective.

## Materials and Methods

### The Game of Darts

Dart players compete in so called ‘legs.’ Scoring in most tournaments is set based, implying that the player who is the first to win three legs, wins the set. The player who is the first to score more than half of a fixed number of sets (e.g., best of *n*) wins the match. Players take turns and throw a series of three darts at each turn. Players start with a fixed number of points (usually 501), and each player tries to reduce that number to 0 as quickly as possible (i.e., in the fewest possible number of darts). In professional darts, players first try to reduce the remaining points as fast as possible by aiming for the triple-20 (which we label ‘T20’) section in the board, occasionally aiming for triple-19 or triple-18. Three consecutive successful throws at triple-20 yield a maximum score of 180. When the player reaches the vicinity of 0 points (164 and below), players no longer aim for triple-20 and instead start aiming for other sections of the board, because the rules of the game dictate that the final dart should land on a double. For example, when 8 points are remaining, the player can win the leg by throwing a dart in the double-4 section of the board.

Note that the ‘intention’ of skilled players (i.e., which section the player is aiming for in a given throw) is typically evident to spectators and commentators alike. Especially highly skilled professional players, such as investigated here, throw in a very predictable fashion and attempt to score as many points as possible on each throw. Although a dart aimed at the triple-20 section may occasionally land in adjacent sections, such as single 20, or triple-5, and thus yield fewer points than intended, the player will continue to attempt to hit the triple-20 section, unless he decides to change strategy (e.g., aiming at triple-19 or -18).

### Data Collection

For the purpose of this study we first identified the world top-10 ranking players (see Appendix 1) as of January 5, 2015. We then selected for each player 8 recent official matches, that were held between 2012 and 2015. Given that we could not find a throw-by-throw record with individual values for each triplet, we decided to visually inspect each match on YouTube (see Appendix 1 for the list of the analyzed matches), which holds complete televised matches. Each match was individually watched and manually scored by one of three students, who were all avid darts fans and highly knowledgeable of the game. The students received clear instructions on how to score the individual throws. Each match was saved in a separate Excel file, which was subsequently used to organize and analyze the data, using Matlab and R. We used a custom made function in R to extract relevant statistics, as described in the “*Analysis”* Section.

### Scoring Procedure

We first classified each throw as successful (‘1’) or unsuccessful (‘0’). More specifically, a dart that landed on T20 was scored as 1, and a dart that was directed at T20 but landed elsewhere (e.g., single 20) was scored as 0. For example, three consecutive successful throws (*hit–hit–hit)* in the T20 would yield 111. As another example, a turn consisting of T20, 20, and T20 was scored as *hit–miss–hit*. The triplets were then converted to independent pairs; 111 was converted to ‘11’ and ’11.’ A *hit–miss–hit* sequence (101) was converted to ‘10’ and ’01,’ etc. This scoring procedure allows us to study three types of transitions: throw1 → throw2, throw2 → throw3, and throw3 → throw1. This latter transition (i.e., between the last throw of a triplet and the first throw of the next triplet) is interspersed with the throws of the opponent, and thus taking up considerably more time than the within-leg transitions. Finally, for each player we counted the occurrence of each of the four so defined combinations. Sequential effects, if present, were expected to take place from throw1 → throw2 and throw2 → throw3, but to be weaker or even non-existent for the pair consisting of throw3 → throw1. The operational choice to define success and failure in terms of hitting T20 is motivated from the fact that the three-dart average of the best players in the world is around 100, which implies that the probability of hitting the T20 is typically higher than 1/3, that is, sufficiently high to make the analysis meaningful. With respect to our data, we found that, averaged over all hits and misses, and averaged over all players, hit (T20) percentage was 43%, which represents overall skill level in this sample.

**Table 1** shows an example involving the match of Adrian Lewis (playing van Barneveld) on December 30th, 2014 (PDC 2015), and **Table 2** shows the match of Phil Taylor (playing van Barneveld) on December 30th, 2012 (PDC 2013).

**Table 1:** Contingency table showing the number of *hit–hit, hit–miss, miss–hit*, and *miss–miss* occurrences by Adrian Lewis (match 32 in Appendixs 1 and 2).

		Throw #2:		
		*Hit(1)*	*Miss(0)*	*Total*
**Throw #1:**	*Hit(1)*	23	14	37
	*Miss(0)*	15	27	42
	*Total*	38	41	79

*Only transitions from the first to the second throw are shown.*

**Table 2:** Contingency table showing the number of *hit–hit, hit–miss, miss–hit*, and *miss–miss* occurrences by Phil Taylor (match 10 in Appendixs 1 and 2).

		Throw #3:		
		*Hit(1)*	*Miss(0)*	*Total*
**Throw #2:**	*Hit(1)*	9	20	29
	*Miss(0)*	8	9	17
	*Total*	17	29	46

*Only transitions from the second to the third throw are shown.*

Note that players predominantly aim for triple-20, but on occasion may switch to triple-18 or triple-19 during a turn, for example because a previously thrown dart, still stuck in the dart board, is blocking the path toward triple-20. In that case the throw after the switch cannot be paired to the previous throw, nor to the following throw (if aimed again at T20). These throws were therefore excluded from the data analysis. In the current analysis, we only analyzed T20 sequences, as throws to T19 or T18 (single or multiple attempts) happened only occasionally.

Since we were dependent on the quality of the video footage of the matches shown on YouTube, not all throwing pairs were registered. If the quality was too low (e.g., due to blur, or if the throw was partially blocked from view), we could not reliably determine the score of the dart, in which case the dart was ignored and not entered into the data sample. The duration of matches varied from 30 min to more than an hour, depending on the performance and how far up in the tournament the match was. Most of the longer during matches were typically divided into several shorter videos on YouTube (part 1, part 2, etc.). In some cases one part of the match was missing such that observations could not be recorded from that part of the match.

Using the procedure described above, a total of 15,187 binary combinations were identified and used for further analysis; 6396 for throw1 → throw2; 4948 for throw2 → throw3; 3843 for throw3 → throw1.

## Analysis

In the present study we investigated whether dart throwing can be regarded as repeated Bernoulli trials (a.k.a. binomial process), with two possible outcomes in each throw, namely hit or miss (as defined above), or whether systematic deviations from the Bernoulli distribution occurred. The probability of success is assumed to be constant (consider flipping a coin) but does not have to be equal to 0.5, depending on the player’s overall skill level. The repeated experiments are assumed to be independent. There are (at least) two scenarios that may account for systematic deviations from the assumptions made above in sequences of dart throws. These scenarios, and their mathematical derivations, have been described in detail by Wardrop (1995) and more recently by Yaari and Eisenmann (2011; see also Yaari and David, 2012), and we will summarize them here.

First, there could be *sequential dependence* between the throws, in that ‘success breeds success’ and ‘failure breeds failure.’ The null hypothesis states that the outcome of a given dart throw is statistically independent from the outcome of the preceding throw. If the proportion of *hit–hit* or *miss–miss* is very high (or very low) relative to the marginal totals this will likely yield significance. Second, it could be that performance is not stable over time, but that the chance of success increases or decreases in the course of the match. That is, there may be substantial fluctuations in the probability of success over the course of performance, which do not have to result from a sensori-motor feedback mechanism. Thus, the probability of success may be *non-stationary*.

Measures of sequential dependence and non-stationarity can be derived by assuming that the entire sequence of (both successful and unsuccessful) dart throws is drawn from a hypergeometric distribution, i.e., sampled without replacement (Yaari and Eisenmann, 2011). With respect to the sequential dependence: the null hypothesis states that success (or failure) in the second throw is statistically independent of success or failure in the first throw. This can be tested using Fisher’s exact test. As an example, consider the contingency table in **Table 1**, showing the number of occurrences of each of the four possible transitions, for one illustrative match, focussing on throw1 → throw2. As can be seen, out of the 79 identified pairs, the number of hit–hit (23) and miss–miss (27) transitions was substantially larger than the number of hit–miss (14) and miss–hit (15) transitions. Indeed, Fisher’s exact test yields a *Z* score of 2.333 and a *p*-value of 0.02, thus lending statistical support to our observation.

With respect to non-stationarity, the null hypothesis states that the number of successful throws at the first or second attempt follows a random sample from the same underlying distribution. Significance was tested using McNemar’s test. As an example, the contingency table in **Table 2** yields a *Z* score of -2.489 and a *p*-value of 0.013; this is due to many hits in the first throw (29 out of 46) compared to the number of hits in the second throw (17 out of 46) of the triplets, which could reflect a performance decrement.

For both measures, the difference between the observed value and the expected value can be assessed statistically using *z*-scores and *p*-values (α = 0.05) to quantify significance. We calculated *z*-scores for each player, testing (a) sequential dependence in the data; we label this *Z*-IND (‘Independence’), and (b) (non)-stationarity, labeled *Z*-NS (‘stationarity’).

Note that we performed this analysis separately for each player, and not for the aggregate (summed total) data, as this may give rise to ‘Simpson’s paradox.’ Simply put, the paradox states that a trend that is visible within one entity (e.g., player) may disappear or even change sign when the data of all entities (e.g., players) are combined into a sum score (e.g., Wardrop, 1995).

## Results

The statistical outcomes (*Z*-IND and *Z*-NS) of all 80 matches are presented in Appendix 2, and allow for the following observations. Focusing on *Z*-IND, we found that throw1 → throw2 yielded 12 sets with significant positive trends (i.e., *Z*-IND > 1.96) and 3 with significant negative trends (i.e., *Z*-IND < -1.96), while the remaining 65 transitions were not significant. A somewhat similar pattern was found for throw2 → throw3: 9 sets with significant positive trends, 2 with significant negative trends, and the remaining 69 transitions were not significant. Finally, focusing on throw3 → throw1, we found 2 transitions with significant positive trends, 3 with significant negative trends, and 75 non-significant transitions. Thus, there is evidence of sequential effects, that is, an excess of ‘11’ and/or ‘00’ patterns, relative to the alternate patterns (‘01’ and ‘10’). Note that this was mainly observed in throw1 → throw2 and to a lesser extent in throw2 → throw3, and hardly in throw3 → throw1.

A notable exception was formed by three matches of Peter Wright (#33, #34, and #35). These matches were characterized by many occurrences of ‘01’ and ’10,’ relative to ‘00’ and ’11.’ This pattern yielded significance with Fisher’s exact test, but with negative *Z*-IND scores. In other words, a miss was more often followed by a hit, and a hit was more often followed by a miss. Inspection of these matches indeed revealed that Wright often threw triplets consisting of T20-20-T20 (‘10’and ‘01’) or 20-T20-20 (‘01’ and ‘10’). Note also that, of the 16 matches of Peter Wright (throw1 → throw2, and throw2 → throw3), 14 yielded negative *Z*-IND scores, four of which reached significance. This suggests a consistent, albeit deviating and highly variable, pattern of player performance.

How can we identify statistical patterns across the 80 matches? As stated, we could simply add all instances of 00, 01, 10, and 11 across players, and analyze the summed scores, but this could lead to Simpson’s Paradox. So instead we decided to count the number of positive and negative *Z*-scores (disregarding the handful of zeroes), and to use a binomial test (at *p* = 0.05; 2-tailed) to test whether these counts are significantly different. For throw1 → throw2 we found 58 positive *Z*-IND scores (out of a total of 79 values), yielding a *p*-value of 2*10^-5^. For throw2 → throw3 we found 46 out of 78 positive *Z*-IND scores, yielding a *p*-value of 0.11, and for throw3 → throw1 we found 41 out of 79 positive *Z*-IND scores, yielding a *p*-value of 0.74. Thus, across the matches there is evidence of statistical dependence from throw1 to throw2 but not for the remaining transitions.

**FIGURE 1:**
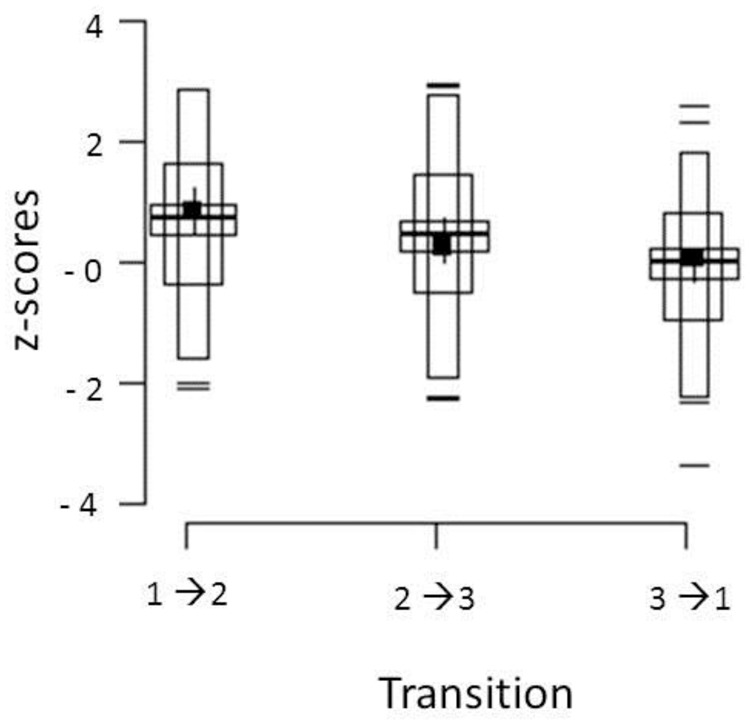
**Distribution of *Z*-IND scores for the three transitions.** Observations lying ± 2 SD beyond the mean are represented by dashes, observations between -2 and +2 SD are represented by the longest and thinnest box. Observations that fall between the mean of the first half of the data and the mean of the second half of the data are represented by the intermediate boxes. The mean of the data is represented by the middle thickest and longest horizontal line. The 95% bootstrap bias-corrected and accelerated CIs (95% CIBCa) are represented by the wide box surrounding the mean line. The median and its 95% CIBCa are represented by a solid small square and whiskers around it (see Marmolejo-Ramos and Tian, 2010, for details).

**Figure 1** shows the distribution of *Z*-IND scores for each of the three transitions. We plotted the scores in so-called shifting boxplots. This manner of graphically representing data was introduced by Marmolejo-Ramos and Tian (2010) and allows researchers to visualize various summary statistics of the distribution such as dispersion and skewness in one plot.

Focusing now on *Z*-NS (stationarity) we first note that the number of significances is much smaller compared to the analysis of *Z*-IND. Overall, there were 21 significances, 17 negative ones and 4 positive ones. Recall that negative *Z*-NS scores are indicative of gradual performance decline over time. Similar to *Z*-IND, we used the binomial test to identify the distribution of positive and negative *Z*-NS scores. For throw1 → throw2 we found 40 out of 78 negative *Z*-NS scores, yielding a *p*-value of 0.82 (two-tailed). For throw2 → throw3 we found 47 out of 77 negative *Z*-NS scores, yielding a *p*-value of 0.054, and for throw3 → throw1 we found 45 out of 77 negative *Z*-NS scores, yielding a *p*-value of 0.14. Thus, across the matches, although no *p*-value was lower than the α = 0.05 threshold, they all showed similar trends indicating that performance declined in the course of the match.

However, combining these three *Z*-scores using Stouffer’s method yielded a significant *p*-value of 0.035, which is in support of this trend.

## Discussion

To our knowledge, the present study is the first to examine whether successive dart throws in professional tournaments exhibit patterns of success and failure that are characterized by statistical independence and stationarity. That is, we tested whether darts performance can be modeled as repeated independent Bernoulli trials with a constant probability of success. To this end, we analyzed the darts performance of 10 professional darts players, collected over 80 matches, focusing exclusively on throws directed at triple-20 and ignoring all other throws.

The analysis revealed that the probability of success of the second throw in a triplet was not statistically independent from the outcome of the preceding (first) throw, as evidenced by 12 (out of 80) significances, and an overall significant effect when combining all *z*-scores. For the other transitions (2 → 3, and 3 → 1) there were significances on an individual level, but not on an aggregate level. A clear exception to this pattern was the throwing behavior of Peter Wright; in seven out of the eight matches his performance sequences for throw1 → throw2 were characterized by negative *Z*-IND scores, three of which were significant (*p* < 0.05). These results pointed to variable throwing behavior within a triplet, such as hit–miss–hit, or miss–hit–miss sequences.

These analyses suggest that the rate of success is not constant but variable in that it may show statistically detectable fluctuations. The present study thus adds to recent evidence in favor of the presence of sequential performance effects in sports and against suggestions that the hot hand effect would be non-existent (Avugos et al., 2013). Or put more formally or succinctly, in professional darts, evidence exists for both sequential dependence, as well as some degree non-stationarity, implying that performance does not, or at least not always, constitute a stationary random independent process.

The question remains how the observed sequential dependence and non-stationarity should be explained. As it stands, we neither know the motor control mechanisms (or coordination dynamics) underlying the observed carry-over effects within triplets nor the possible causes of non-stationarity. Given this state of affairs, we do not wish to speculate too much about these mechanisms and causes, but with regard to the observed sequential dependence it is tempting to hypothesize that some sort of motor ‘memory’ and/or sensori-motor feedback mechanism is involved. It would be interesting to examine this possibility further, for instance by taking the time interval between successive dart throws within a triplet into account in future analyses, perhaps in combination with a detailed analyses of the kinematics of the throwing action and the precise landing positions of the throws. It could well be that the strength of the observed sequential performance effect is a function of the time interval between throws, but quite some statistical power is probably needed to test this hypothesis. Another possibility would be to manipulate the interval between successive dart throws experimentally by cueing them.

As yet another possible avenue for future research, it is worth noting that most of the variability in darts performance tends to occur along the vertical dimension, and much less so in the horizontal dimension (e.g., Smeets et al., 2002). Professional darts players tend to fixate the configuration of their shoulder and elbow angles, thereby reducing spatial errors in the left–right direction. However, there is considerable variability in timing, especially in terms of the moment of release. These timing fluctuations will result in spatial fluctuations of the position of the dart in the vertical plane. Thus, a dart, directed at T20 will, due to limitations in timing precision, tend to land below or above the T20 region. It could be that sequential effects in performance as observed in our study originate from autocorrelations in the neural control of timing processes. It should be noted that we adopted a narrow definition of ‘performance’ in that we focus exclusively on hits/misses aimed at triple-20. All other throws, such as compulsory doubles, and strategic decisions to aim for other sections of the board, are of course greatly important for overall darts performance, but these factors were not taken into account here and could perhaps be included in future research.

In closing we would like to highlight a final limitation of this study: we focused on individual performances only without taking the performance of the opponent into account. It goes without saying that performance levels are strongly influenced by the actions of the opponent, such that factors like arousal, effort, concentration, ‘choking’, and fatigue all play into darts performance. It seems reasonable to assume that factors such as these are responsible for the observed weak non-stationarity, but given the multitude of possible influences this seems even harder to disentangle further than the observed sequential dependence between throws.

## Author Contributions

JS, GY and PB authored the document. KW and JB conceived the study and helped in analyzing the data.

## Conflict of Interest Statement

The authors declare that the research was conducted in the absence of any commercial or financial relationships that could be construed as a potential conflict of interest.

## Footnotes

1 In the present study, we will not consider long range correlations in performance, even though there is substantial evidence in the motor control literature that motor output, such as the control of locomotion, can exhibit ‘fractal’ behavior.

2 Likewise, one could contend that the dynamical organization of the dart throw is governed from throw to throw by the same parameter and graph dynamics (Saltzman and Munhall, 1992), but in this explanation it is left implicit how movement parameters are preserved over consecutive attempts.
