# Supplementary material for: Evidence for Sequential Performance Effects in Professional Darts
Source: Front Psychol. 2018 Apr 26;9:591. doi: 10.3389/fpsyg.2018.00591 (PMC5932194; doi:10.3389/fpsyg.2018.00591)
Supplement: Supplementary file 1 [file Data_Sheet_1.docx]

**Appendix 1**

Details of the analysed matches of each player.

| nr. | Player | Opponent | Tournament | Round | Date (dd/mm/yyyy) |
| --- | --- | --- | --- | --- | --- |
|  |  |  |  |  |  |
| 1 | Van Gerwen | V Barneveld | GSD 2012 | Final | 18/11/2012 |
| 2 |  | Lewis | PDC 2014 | Semi final | 30/12/2013 |
| 3 |  | Wright | PDC 2014 | Final | 01/01/2014 |
| 4 |  | Whitlock | WMP 2014 | Semi final | 26/07/2014 |
| 5 |  | Taylor | WMP 2014 | Final | 27/07/2014 |
| 6 |  | Wade | WGP 2014 | Final | 12/10/2014 |
| 7 |  | Thornton | PDC 2015 | Quarter final | 01/01/2015 |
| 8 |  | Anderson | PDC 2015 | Semi final | 03/01/2015 |
|  |  |  |  |  |  |
| 9 | Phil Taylor | Wade | WMP 2012 | Final | 29/07/2012 |
| 10 |  | V Barneveld | PDC 2013 | Semi final | 30/12/2012 |
| 11 |  | Lewis | GSD 2013 | Semi final | 17/11/2013 |
| 12 |  | Anderson | WMP 2014 | Semi final | 26/07/2014 |
| 13 |  | V Gerwen | WMP 2014 | Final | 27/07/2014 |
| 14 |  | Chisnall | GSD 2014 | Final | 16/11/2014 |
| 15 |  | V Barneveld | PDC 2015 | Semi final | 03/01/2015 |
| 16 |  | Anderson | PDC 2015 | Final | 04/01/2015 |
|  |  |  |  |  |  |
| 17 | Gary Anderson | Lewis | WMP 2014 | Quarter | 25/07/2014 |
| 18 |  | Taylor | WMP 2014 | Semi final | 26/07/2014 |
| 19 |  | Wade | WGP 2014 | Semi final | 11/10/2014 |
| 20 |  | Lewis | PDC P. 2014 | Final | 30/11/2014 |
| 21 |  | Reyes | PDC 2015 | 3rd | 30/12/2014 |
| 22 |  | Wright | PDC 2015 | Quarter final | 01/01/2015 |
| 23 |  | V Gerwen | PDC 2015 | Semi final | 03/01/2015 |
| 24 |  | Taylor | PDC 2015 | Final | 04/01/2015 |
|  |  |  |  |  |  |
| 25 | Adrian Lewis | V Gerwen | PDC 2013 | Quarter final | 29/12/2012 |
| 26 |  | Taylor | GSD 2013 | Semi final | 17/11/2013 |
| 27 |  | Wade | PDC 2014 | Quarter final | 29/12/2013 |
| 28 |  | V Gerwen | PDC 2014 | Semi final | 30/12/2013 |
| 29 |  | Anderson | WMP 2014 | Quarter final | 25/07/2014 |
| 30 |  | Anderson | PDC P. 2014 | Final | 30/11/2014 |
| 31 |  | Brown | PDC 2015 | 2nd | 28/12/2014 |
| 32 |  | V Barneveld | PDC 2015 | 3rd | 30/12/2014 |
|  |  |  |  |  |  |
| 33 | Peter Wright | Laursen | PDC 2014 | 2nd | 20/12/2013 |
| 34 |  | Smith | PDC 2014 | 3rd | 27/12/2013 |
| 35 |  | Newton | PDC 2014 | Quarter final | 28/12/2013 |
| 36 |  | Whitlock | PDC 2014 | Semi final | 30/12/2013 |
| 37 |  | V Gerwen | PDC 2014 | Final | 01/01/2014 |
| 38 |  | Huybrechts | PDC 2015 | 2nd | 27/12/2014 |
| 39 |  | Hamilton | PDC 2015 | 3rd | 30/12/2014 |
| 40 |  | Anderson | PDC 2015 | Quarter final | 01/01/2015 |
|  |  |  |  |  |  |
| 41 | James Wade | Taylor | WMP 2012 | Final | 29/07/2012 |
| 42 |  | V Gerwen | PDC 2013 | Semi final | 30/12/2012 |
| 43 |  | Lewis | PDC 2014 | Quarter final | 29/12/2013 |
| 44 |  | Whitlock | WMP 2014 | Quarter final | 24/07/2014 |
| 45 |  | Thornton | WGP 2014 | 2nd | 08/10/2014 |
| 46 |  | Taylor | WGP 2014 | Quarter final | 10/10/2014 |
| 47 |  | Anderson | WGP 2014 | Semi final | 11/10/2014 |
| 48 |  | V Gerwen | WGP 2014 | Final | 12/10/2014 |
|  |  |  |  |  |  |
| 49 | Simon Whitlock | Chisnall | PDC 2013 | 3rd | 27/12/2012 |
| 50 |  | V Barneveld | PDC 2013 | Quarter final | 28/12/2012 |
| 51 |  | Painter | PDC 2014 | 3rd | 27/12/2013 |
| 52 |  | White | PDC 2014 | Quarter final | 28/12/2013 |
| 53 |  | Wright | PDC 2014 | Semi final | 30/12/2013 |
| 54 |  | V Barneveld | WMP 2014 | 2nd | 22/07/2014 |
| 55 |  | Wade | WMP 2014 | Quarter final | 24/07/2014 |
| 56 |  | V Gerwen | WMP 2014 | Semi final | 26/07/2014 |
|  |  |  |  |  |  |
| 57 | Robert Thornton | Nicholson | PDC 2013 | 2nd | 21/12/2012 |
| 58 |  | Taylor | GSD 2013 | Finale | 17/11/2013 |
| 59 |  | Beau Anderson | PDC 2014 | 2nd | 21/12/2013 |
| 60 |  | Wade | WGP 2014 | 2nd | 08/10/2014 |
| 61 |  | Chisnall | Masters 2015 | 1st | 01/11/2014 |
| 62 |  | Baxter | PDC 2015 | 2nd | 27/12/2014 |
| 63 |  | Van de Pas | PDC 2015 | 3rd | 29/12/2014 |
| 64 |  | V Gerwen | PDC 2015 | Quarter final | 01/01/2015 |
|  |  |  |  |  |  |
| 65 | Dave Chisnall | Whitlock | PDC 2013 | 3rd | 27/12/2012 |
| 66 |  | V Gerwen | WGP 2013 | Quarter final | 11/10/2013 |
| 67 |  | Pipe | WGP 2013 | Semi final | 12/10/2013 |
| 68 |  | Hamilton | WMP 2014 | 2nd | 22/07/2014 |
| 69 |  | V Gerwen | WMP 2014 | Quarter final | 24/07/2014 |
| 70 |  | Thornton | Masters 2015 | 1st | 01/11/2014 |
| 71 |  | K Huybrechts | GSD 2014 | Semi final | 16/11/2014 |
| 72 |  | Taylor | GSD 2014 | Final | 16/11/2014 |
|  |  |  |  |  |  |
| 73 | R. van Barneveld | V Gerwen | GSD 2012 | Final | 18/11/2012 |
| 74 |  | Whitlock | PDC 2013 | Quarter final | 28/12/2012 |
| 75 |  | Taylor | PDC 2013 | Semi final | 30/12/2012 |
| 76 |  | Whitlock | WMP 2014 | 2nd | 22/07/2014 |
| 77 |  | Caven | PDC 2015 | 2nd | 29/12/2014 |
| 78 |  | Lewis | PDC 2015 | 3rd | 30/12/2014 |
| 79 |  | Bunting | PDC 2015 | Quarter final | 02/01/2015 |
| 80 |  | Taylor | PDC 2015 | Semi final | 03/01/2015 |

*Note:* PDC: PDC World Darts Championship; WMP: World Match Play; WGP: World Grand Prix; GSD: Grand Slam of Darts; PDC P.: PDC Players Championship finals; Masters: The Masters.

**Appendix 2**

Statistical outcomes of the 80 matches, for throw 1 🡪 throw 2, presented in the same order as in Appendix 1. Significant values (p < .05) are presented in bold typeface.

| nr. | HH | HM | MH | MM | z-IND | p-IND | Z-NS | p-NS |
| --- | --- | --- | --- | --- | --- | --- | --- | --- |
|  |  |  |  |  |  |  |  |  |
| 1 | 20 | 13 | 13 | 27 | 2.385 | **0.017** | 0 | 1 |
| 2 | 20 | 13 | 15 | 20 | 1.453 | 0.146 | 0.342 | 0.733 |
| 3 | 36 | 23 | 21 | 26 | 1.668 | 0.095 | -0.275 | 0.783 |
| 4 | 21 | 22 | 8 | 25 | 2.173 | **0.03** | -2.267 | **0.023** |
| 5 | 21 | 19 | 10 | 19 | 1.474 | 0.14 | -1.527 | 0.127 |
| 6 | 25 | 16 | 9 | 13 | 1.511 | 0.131 | -1.265 | 0.206 |
| 7 | 33 | 17 | 12 | 20 | 2.514 | **0.012** | -0.788 | 0.43 |
| 8 | 27 | 21 | 18 | 27 | 1.559 | 0.119 | -0.439 | 0.661 |
|  |  |  |  |  |  |  |  |  |
| 9 | 24 | 18 | 16 | 21 | 1.225 | 0.22 | -0.317 | 0.751 |
| 10 | 25 | 26 | 19 | 24 | 0.465 | 0.642 | -1.018 | 0.308 |
| 11 | 22 | 13 | 22 | 13 | 0 | 1 | 1.529 | 0.126 |
| 12 | 25 | 20 | 22 | 15 | -0.353 | 0.724 | 0.314 | 0.754 |
| 13 | 18 | 21 | 14 | 20 | 0.425 | 0.671 | -1.155 | 0.248 |
| 14 | 15 | 21 | 22 | 21 | -0.837 | 0.403 | 0.159 | 0.874 |
| 15 | 26 | 17 | 25 | 30 | 1.468 | 0.142 | 1.141 | 0.254 |
| 16 | 38 | 27 | 29 | 42 | 2.045 | **0.041** | 0.242 | 0.809 |
|  |  |  |  |  |  |  |  |  |
| 17 | 15 | 22 | 14 | 17 | -0.381 | 0.703 | -1.368 | 0.171 |
| 18 | 26 | 17 | 23 | 23 | 0.986 | 0.324 | 0.897 | 0.369 |
| 19 | 23 | 14 | 13 | 15 | 1.254 | 0.21 | -0.176 | 0.86 |
| 20 | 15 | 10 | 5 | 15 | 2.322 | **0.02** | -1.048 | 0.295 |
| 21 | 15 | 12 | 13 | 14 | 0.54 | 0.589 | 0.192 | 0.848 |
| 22 | 18 | 18 | 21 | 29 | 0.731 | 0.465 | 0.46 | 0.646 |
| 23 | 28 | 29 | 24 | 36 | 0.988 | 0.323 | -0.654 | 0.513 |
| 24 | 34 | 32 | 36 | 46 | 0.919 | 0.358 | 0.466 | 0.641 |
|  |  |  |  |  |  |  |  |  |
| 25 | 32 | 20 | 16 | 29 | 2.539 | **0.011** | -0.573 | 0.567 |
| 26 | 34 | 13 | 9 | 12 | 2.312 | **0.021** | -0.722 | 0.47 |
| 27 | 20 | 13 | 18 | 14 | 0.354 | 0.724 | 0.877 | 0.38 |
| 28 | 13 | 17 | 21 | 24 | -0.282 | 0.778 | 0.658 | 0.51 |
| 29 | 11 | 14 | 12 | 28 | 1.14 | 0.254 | -0.362 | 0.717 |
| 30 | 10 | 9 | 14 | 19 | 0.704 | 0.481 | 0.991 | 0.322 |
| 31 | 21 | 23 | 11 | 20 | 1.049 | 0.294 | -1.953 | 0.051 |
| 32 | 23 | 14 | 15 | 27 | 2.333 | **0.02** | 0.159 | 0.874 |
|  |  |  |  |  |  |  |  |  |
| 33 | 12 | 26 | 27 | 23 | -2.085 | **0.037** | 0.152 | 0.88 |
| 34 | 20 | 32 | 34 | 22 | -2.3 | **0.021** | 0.272 | 0.786 |
| 35 | 21 | 44 | 39 | 41 | -1.992 | **0.046** | -0.592 | 0.554 |
| 36 | 16 | 30 | 31 | 27 | -1.89 | 0.059 | 0.139 | 0.889 |
| 37 | 21 | 45 | 38 | 49 | -1.488 | 0.137 | -0.813 | 0.416 |
| 38 | 13 | 16 | 24 | 23 | -0.525 | 0.6 | 1.305 | 0.192 |
| 39 | 12 | 14 | 15 | 12 | -0.678 | 0.498 | 0.193 | 0.847 |
| 40 | 16 | 20 | 19 | 47 | 1.584 | 0.113 | -0.147 | 0.883 |
|  |  |  |  |  |  |  |  |  |
| 41 | 21 | 29 | 23 | 35 | 0.246 | 0.806 | -0.822 | 0.411 |
| 42 | 8 | 22 | 27 | 46 | -1 | 0.317 | 0.748 | 0.455 |
| 43 | 7 | 18 | 22 | 24 | -1.612 | 0.107 | 0.689 | 0.491 |
| 44 | 11 | 14 | 29 | 31 | -0.363 | 0.717 | 2.36 | **0.018** |
| 45 | 9 | 14 | 12 | 29 | 0.8 | 0.424 | -0.371 | 0.711 |
| 46 | 8 | 13 | 10 | 15 | -0.13 | 0.896 | -0.629 | 0.529 |
| 47 | 14 | 21 | 18 | 19 | -0.733 | 0.464 | -0.499 | 0.617 |
| 48 | 12 | 22 | 19 | 33 | -0.117 | 0.907 | -0.47 | 0.638 |
|  |  |  |  |  |  |  |  |  |
| 49 | 11 | 16 | 14 | 25 | 0.396 | 0.692 | -0.355 | 0.723 |
| 50 | 17 | 12 | 12 | 25 | 2.111 | **0.035** | 0 | 1 |
| 51 | 9 | 4 | 11 | 17 | 1.763 | 0.078 | 1.567 | 0.117 |
| 52 | 28 | 20 | 22 | 31 | 1.68 | 0.093 | 0.281 | 0.779 |
| 53 | 14 | 20 | 16 | 23 | 0.013 | 0.99 | -0.665 | 0.506 |
| 54 | 14 | 10 | 11 | 20 | 1.672 | 0.094 | 0.191 | 0.849 |
| 55 | 19 | 19 | 6 | 28 | 2.859 | **0.004** | -2.176 | **0.03** |
| 56 | 15 | 16 | 14 | 31 | 1.514 | 0.13 | -0.331 | 0.741 |
|  |  |  |  |  |  |  |  |  |
| 57 | 21 | 20 | 34 | 43 | 0.729 | 0.466 | 1.851 | 0.064 |
| 58 | 9 | 18 | 15 | 26 | -0.273 | 0.785 | -0.529 | 0.597 |
| 59 | 11 | 9 | 8 | 17 | 1.535 | 0.125 | -0.212 | 0.832 |
| 60 | 10 | 9 | 16 | 18 | 0.385 | 0.7 | 1.369 | 0.171 |
| 61 | 8 | 9 | 10 | 16 | 0.552 | 0.581 | 0.218 | 0.827 |
| 62 | 10 | 6 | 12 | 17 | 1.342 | 0.18 | 1.273 | 0.203 |
| 63 | 10 | 15 | 17 | 16 | -0.863 | 0.388 | 0.372 | 0.71 |
| 64 | 25 | 19 | 21 | 26 | 1.151 | 0.25 | 0.296 | 0.767 |
|  |  |  |  |  |  |  |  |  |
| 65 | 13 | 25 | 18 | 29 | -0.387 | 0.699 | -1.09 | 0.276 |
| 66 | 16 | 7 | 10 | 10 | 1.294 | 0.196 | 0.65 | 0.516 |
| 67 | 12 | 8 | 15 | 13 | 0.438 | 0.661 | 1.422 | 0.155 |
| 68 | 16 | 12 | 13 | 22 | 1.57 | 0.116 | 0.178 | 0.859 |
| 69 | 21 | 16 | 12 | 32 | 2.674 | **0.008** | -0.632 | 0.527 |
| 70 | 10 | 9 | 8 | 9 | 0.329 | 0.742 | -0.234 | 0.815 |
| 71 | 19 | 12 | 23 | 22 | 0.871 | 0.384 | 1.78 | 0.075 |
| 72 | 18 | 12 | 18 | 16 | 0.564 | 0.573 | 1.057 | 0.291 |
|  |  |  |  |  |  |  |  |  |
| 73 | 21 | 20 | 26 | 43 | 1.382 | 0.167 | 0.824 | 0.41 |
| 74 | 25 | 20 | 10 | 29 | 2.757 | **0.006** | -1.54 | 0.124 |
| 75 | 21 | 34 | 33 | 57 | 0.182 | 0.855 | -0.121 | 0.904 |
| 76 | 9 | 19 | 10 | 31 | 0.703 | 0.482 | -1.611 | 0.107 |
| 77 | 16 | 12 | 20 | 37 | 1.923 | 0.055 | 1.263 | 0.207 |
| 78 | 13 | 22 | 18 | 30 | -0.033 | 0.974 | -0.632 | 0.527 |
| 79 | 21 | 34 | 15 | 26 | 0.159 | 0.874 | -2.739 | **0.006** |
| 80 | 24 | 22 | 21 | 37 | 1.624 | 0.104 | -0.139 | 0.889 |

*Note:* HH: number of hit-hit; HM: number of hit-miss; MH: number of miss-hit; MM: number of miss-miss; z: z-score; p: p-value; IND: independence; NS: non-stationarity

**Appendix 2 (continued)**

Statistical outcomes of the 80 matches, for throw 2 🡪 throw 3, presented in the same order as in Appendix 1. Significant values (p < .05) are presented in bold typeface.

| nr. | HH | HM | MH | MM | z-IND | p-IND | Z-NS | p-NS |
| --- | --- | --- | --- | --- | --- | --- | --- | --- |
|  |  |  |  |  |  |  |  |  |
| 1 | 14 | 10 | 9 | 12 | 1.025 | 0.306 | -0.21 | 0.834 |
| 2 | 10 | 15 | 6 | 10 | 0.158 | 0.874 | -1.976 | **0.048** |
| 3 | 26 | 22 | 12 | 9 | -0.227 | 0.82 | -1.75 | 0.08 |
| 4 | 10 | 12 | 14 | 16 | -0.086 | 0.932 | 0.393 | 0.694 |
| 5 | 15 | 9 | 5 | 19 | 2.897 | **0.004** | -0.815 | 0.415 |
| 6 | 14 | 16 | 16 | 9 | -1.274 | 0.203 | 0 | 1 |
| 7 | 21 | 11 | 11 | 11 | 1.138 | 0.255 | 0 | 1 |
| 8 | 16 | 20 | 15 | 7 | -1.743 | 0.081 | -0.936 | 0.349 |
|  |  |  |  |  |  |  |  |  |
| 9 | 12 | 17 | 13 | 14 | -0.505 | 0.614 | -0.753 | 0.451 |
| 10 | 9 | 20 | 8 | 9 | -1.075 | 0.282 | -2.489 | **0.013** |
| 11 | 22 | 12 | 6 | 7 | 1.147 | 0.251 | -1.299 | 0.194 |
| 12 | 16 | 17 | 10 | 10 | -0.106 | 0.916 | -1.362 | 0.173 |
| 13 | 15 | 8 | 11 | 10 | 0.855 | 0.392 | 0.64 | 0.522 |
| 14 | 16 | 11 | 13 | 10 | 0.194 | 0.847 | 0.401 | 0.688 |
| 15 | 14 | 27 | 15 | 18 | -0.984 | 0.325 | -1.969 | 0.**049** |
| 16 | 26 | 25 | 27 | 20 | -0.638 | 0.523 | 0.286 | 0.775 |
|  |  |  |  |  |  |  |  |  |
| 17 | 10 | 16 | 17 | 9 | -1.924 | 0.054 | 0.195 | 0.845 |
| 18 | 22 | 19 | 10 | 12 | 0.616 | 0.538 | -1.618 | 0.106 |
| 19 | 21 | 14 | 7 | 13 | 1.768 | 0.077 | -1.343 | 0.179 |
| 20 | 9 | 7 | 10 | 9 | 0.211 | 0.833 | 0.712 | 0.476 |
| 21 | 16 | 10 | 14 | 6 | -0.591 | 0.555 | 0.85 | 0.395 |
| 22 | 25 | 10 | 14 | 16 | 2.016 | **0.044** | 0.706 | 0.48 |
| 23 | 24 | 21 | 22 | 26 | 0.719 | 0.472 | 0.146 | 0.884 |
| 24 | 33 | 29 | 27 | 26 | 0.243 | 0.808 | -0.264 | 0.792 |
|  |  |  |  |  |  |  |  |  |
| 25 | 18 | 21 | 22 | 18 | -0.781 | 0.435 | 0.159 | 0.874 |
| 26 | 22 | 12 | 4 | 12 | 2.595 | **0.009** | -1.625 | 0.104 |
| 27 | 15 | 18 | 10 | 12 | 0 | 1 | -1.521 | 0.128 |
| 28 | 19 | 8 | 19 | 11 | 0.558 | 0.577 | 2.072 | **0.038** |
| 29 | 8 | 10 | 11 | 16 | 0.244 | 0.807 | 0.213 | 0.831 |
| 30 | 6 | 15 | 5 | 19 | 0.596 | 0.551 | -2.19 | **0.029** |
| 31 | 13 | 11 | 15 | 12 | -0.099 | 0.922 | 0.788 | 0.43 |
| 32 | 22 | 15 | 8 | 18 | 2.227 | **0.026** | -1.245 | 0.213 |
|  |  |  |  |  |  |  |  |  |
| 33 | 8 | 22 | 17 | 14 | -2.218 | **0.027** | -0.906 | 0.365 |
| 34 | 17 | 27 | 19 | 19 | -1.028 | 0.304 | -1.246 | 0.213 |
| 35 | 14 | 35 | 23 | 39 | -0.942 | 0.346 | -1.65 | 0.099 |
| 36 | 17 | 23 | 17 | 28 | 0.441 | 0.659 | -0.925 | 0.355 |
| 37 | 16 | 28 | 24 | 39 | -0.181 | 0.856 | -0.559 | 0.576 |
| 38 | 12 | 15 | 14 | 17 | -0.054 | 0.957 | -0.186 | 0.853 |
| 39 | 9 | 10 | 9 | 7 | -0.516 | 0.606 | -0.238 | 0.812 |
| 40 | 9 | 21 | 25 | 33 | -1.19 | 0.234 | 0.625 | 0.532 |
|  |  |  |  |  |  |  |  |  |
| 41 | 13 | 21 | 22 | 30 | -0.374 | 0.709 | 0.155 | 0.877 |
| 42 | 11 | 16 | 19 | 35 | 0.485 | 0.628 | 0.492 | 0.623 |
| 43 | 15 | 13 | 14 | 17 | 0.64 | 0.522 | 0.183 | 0.854 |
| 44 | 16 | 18 | 17 | 18 | -0.125 | 0.901 | -0.17 | 0.865 |
| 45 | 5 | 14 | 27 | 20 | -2.274 | **0.023** | 2.315 | **0.021** |
| 46 | 9 | 8 | 8 | 18 | 1.437 | 0.151 | 0 | 1 |
| 47 | 13 | 21 | 14 | 19 | -0.347 | 0.729 | -1.21 | 0.226 |
| 48 | 22 | 14 | 16 | 38 | 2.946 | **0.003** | 0.302 | 0.763 |
|  |  |  |  |  |  |  |  |  |
| 49 | 11 | 7 | 12 | 17 | 1.301 | 0.193 | 1.034 | 0.301 |
| 50 | 17 | 8 | 16 | 17 | 1.473 | 0.141 | 1.479 | 0.139 |
| 51 | 10 | 8 | 3 | 10 | 1.779 | 0.075 | -1.26 | 0.208 |
| 52 | 26 | 23 | 7 | 25 | 2.775 | **0.006** | -2.507 | **0.012** |
| 53 | 15 | 17 | 19 | 13 | -0.994 | 0.32 | 0.352 | 0.725 |
| 54 | 11 | 11 | 7 | 14 | 1.094 | 0.274 | -0.86 | 0.39 |
| 55 | 15 | 10 | 8 | 23 | 2.562 | **0.01** | -0.38 | 0.704 |
| 56 | 17 | 13 | 12 | 14 | 0.778 | 0.436 | -0.188 | 0.851 |
|  |  |  |  |  |  |  |  |  |
| 57 | 26 | 27 | 12 | 39 | 2.69 | **0.007** | -2.092 | **0.036** |
| 58 | 10 | 14 | 20 | 18 | -0.835 | 0.404 | 1.082 | 0.279 |
| 59 | 11 | 8 | 11 | 14 | 0.903 | 0.367 | 0.637 | 0.524 |
| 60 | 16 | 16 | 11 | 21 | 1.256 | 0.209 | -0.883 | 0.377 |
| 61 | 8 | 9 | 6 | 15 | 1.159 | 0.246 | -0.696 | 0.487 |
| 62 | 13 | 6 | 10 | 8 | 0.796 | 0.426 | 0.932 | 0.351 |
| 63 | 11 | 15 | 11 | 15 | 0 | 1 | -0.783 | 0.434 |
| 64 | 22 | 17 | 14 | 23 | 1.61 | 0.107 | -0.485 | 0.628 |
|  |  |  |  |  |  |  |  |  |
| 65 | 16 | 13 | 12 | 24 | 1.754 | 0.079 | -0.176 | 0.86 |
| 66 | 18 | 12 | 4 | 9 | 1.741 | 0.082 | -1.754 | 0.079 |
| 67 | 19 | 9 | 11 | 10 | 1.089 | 0.276 | 0.409 | 0.683 |
| 68 | 16 | 12 | 7 | 15 | 1.766 | 0.077 | -0.995 | 0.32 |
| 69 | 12 | 19 | 15 | 24 | 0.021 | 0.983 | -0.684 | 0.494 |
| 70 | 9 | 7 | 9 | 4 | -0.704 | 0.481 | 0.529 | 0.597 |
| 71 | 19 | 25 | 8 | 16 | 0.787 | 0.431 | -2.908 | **0.004** |
| 72 | 16 | 19 | 8 | 11 | 0.253 | 0.801 | -2.116 | **0.034** |
|  |  |  |  |  |  |  |  |  |
| 73 | 12 | 24 | 15 | 27 | -0.219 | 0.827 | -1.464 | 0.143 |
| 74 | 14 | 16 | 17 | 15 | -0.504 | 0.614 | 0.179 | 0.858 |
| 75 | 21 | 25 | 28 | 33 | -0.026 | 0.98 | 0.412 | 0.68 |
| 76 | 5 | 12 | 11 | 26 | -0.024 | 0.981 | -0.208 | 0.835 |
| 77 | 17 | 15 | 9 | 16 | 1.277 | 0.202 | -1.119 | 0.263 |
| 78 | 9 | 20 | 12 | 24 | -0.195 | 0.845 | -1.437 | 0.151 |
| 79 | 20 | 11 | 16 | 17 | 1.282 | 0.2 | 0.881 | 0.378 |
| 80 | 25 | 14 | 11 | 23 | 2.688 | **0.007** | -0.495 | 0.621 |

*Note:* HH: number of hit-hit; HM: number of hit-miss; MH: number of miss-hit; MM: number of miss-miss; z: z-score; p: p-value; IND: independence; NS: non-stationarity

**Appendix 2 (continued)**

Statistical outcomes of the 80 matches, for throw 3 🡪 throw 1, presented in the same order as in Appendix 1. Significant values (p < .05) are presented in bold typeface.

| nr. | HH | HM | MH | MM | z-IND | p-IND | Z-NS | p-NS |
| --- | --- | --- | --- | --- | --- | --- | --- | --- |
|  |  |  |  |  |  |  |  |  |
| 1 | 9 | 9 | 5 | 10 | 0.95 | 0.342 | -0.978 | 0.328 |
| 2 | 5 | 10 | 7 | 15 | 0.095 | 0.924 | -0.72 | 0.472 |
| 3 | 10 | 24 | 9 | 15 | -0.641 | 0.522 | -2.784 | **0.005** |
| 4 | 8 | 12 | 10 | 17 | 0.204 | 0.838 | -0.418 | 0.676 |
| 5 | 6 | 8 | 10 | 16 | 0.267 | 0.789 | 0.459 | 0.646 |
| 6 | 11 | 14 | 14 | 9 | -1.157 | 0.247 | 0 | 1 |
| 7 | 6 | 17 | 10 | 8 | -1.896 | 0.058 | -1.538 | 0.124 |
| 8 | 11 | 13 | 12 | 13 | -0.15 | 0.88 | -0.201 | 0.841 |
|  |  |  |  |  |  |  |  |  |
| 9 | 5 | 16 | 15 | 11 | -2.311 | **0.021** | -0.207 | 0.836 |
| 10 | 3 | 10 | 10 | 16 | -0.948 | 0.343 | 0 | 1 |
| 11 | 7 | 15 | 9 | 8 | -1.313 | 0.189 | -1.35 | 0.177 |
| 12 | 6 | 9 | 13 | 8 | -1.28 | 0.201 | 0.938 | 0.348 |
| 13 | 10 | 10 | 4 | 6 | 0.509 | 0.611 | -1.55 | 0.121 |
| 14 | 8 | 13 | 4 | 13 | 0.948 | 0.343 | -2.069 | **0.039** |
| 15 | 14 | 8 | 10 | 25 | 2.587 | **0.01** | 0.38 | 0.704 |
| 16 | 16 | 24 | 17 | 14 | -1.235 | 0.217 | -1.171 | 0.242 |
|  |  |  |  |  |  |  |  |  |
| 17 | 7 | 12 | 6 | 15 | 0.551 | 0.582 | -1.361 | 0.174 |
| 18 | 10 | 11 | 8 | 19 | 1.264 | 0.206 | -0.62 | 0.535 |
| 19 | 9 | 10 | 14 | 8 | -1.034 | 0.301 | 0.878 | 0.38 |
| 20 | 8 | 6 | 5 | 9 | 1.116 | 0.264 | -0.265 | 0.791 |
| 21 | 4 | 19 | 8 | 2 | -3.384 | **0.001** | -2.692 | **0.007** |
| 22 | 9 | 21 | 6 | 14 | 0 | 1 | -3 | **0.003** |
| 23 | 14 | 16 | 20 | 13 | -1.1 | 0.271 | 0.71 | 0.478 |
| 24 | 15 | 27 | 20 | 24 | -0.914 | 0.361 | -1.07 | 0.285 |
|  |  |  |  |  |  |  |  |  |
| 25 | 14 | 19 | 9 | 22 | 1.107 | 0.268 | -1.775 | 0.076 |
| 26 | 11 | 11 | 10 | 12 | 0.298 | 0.765 | -0.212 | 0.832 |
| 27 | 7 | 11 | 11 | 22 | 0.393 | 0.694 | 0 | 1 |
| 28 | 8 | 23 | 5 | 13 | -0.149 | 0.881 | -3.637 | **0** |
| 29 | 5 | 12 | 7 | 16 | -0.069 | 0.945 | -1.156 | 0.248 |
| 30 | 3 | 6 | 7 | 23 | 0.595 | 0.552 | 0.262 | 0.793 |
| 31 | 12 | 16 | 11 | 7 | -1.195 | 0.232 | -1.043 | 0.297 |
| 32 | 6 | 9 | 7 | 24 | 1.217 | 0.224 | -0.451 | 0.652 |
|  |  |  |  |  |  |  |  |  |
| 33 | 8 | 12 | 11 | 18 | 0.145 | 0.885 | -0.205 | 0.837 |
| 34 | 16 | 13 | 16 | 22 | 1.053 | 0.292 | 0.518 | 0.604 |
| 35 | 14 | 11 | 26 | 36 | 1.184 | 0.236 | 2.344 | **0.019** |
| 36 | 11 | 15 | 17 | 25 | 0.148 | 0.882 | 0.349 | 0.727 |
| 37 | 15 | 17 | 28 | 29 | -0.202 | 0.84 | 1.665 | 0.096 |
| 38 | 7 | 13 | 9 | 20 | 0.288 | 0.773 | -0.834 | 0.404 |
| 39 | 4 | 9 | 9 | 7 | -1.348 | 0.178 | 0 | 1 |
| 40 | 8 | 17 | 16 | 27 | -0.43 | 0.667 | -0.178 | 0.859 |
|  |  |  |  |  |  |  |  |  |
| 41 | 8 | 15 | 19 | 25 | -0.661 | 0.509 | 0.712 | 0.477 |
| 42 | 5 | 16 | 14 | 31 | -0.606 | 0.545 | -0.377 | 0.706 |
| 43 | 8 | 13 | 8 | 15 | 0.226 | 0.822 | -1.074 | 0.283 |
| 44 | 9 | 15 | 6 | 22 | 1.263 | 0.207 | -1.814 | 0.07 |
| 45 | 8 | 20 | 11 | 22 | -0.397 | 0.691 | -1.667 | 0.095 |
| 46 | 7 | 6 | 11 | 16 | 0.771 | 0.441 | 1.14 | 0.254 |
| 47 | 13 | 10 | 18 | 19 | 0.588 | 0.556 | 1.462 | 0.144 |
| 48 | 11 | 22 | 19 | 32 | -0.364 | 0.716 | -0.477 | 0.634 |
|  |  |  |  |  |  |  |  |  |
| 49 | 5 | 11 | 5 | 13 | 0.219 | 0.827 | -1.486 | 0.137 |
| 50 | 9 | 10 | 6 | 14 | 1.1 | 0.271 | -0.907 | 0.364 |
| 51 | 1 | 8 | 3 | 13 | -0.49 | 0.624 | -1.596 | 0.111 |
| 52 | 11 | 9 | 15 | 23 | 1.12 | 0.263 | 1.134 | 0.257 |
| 53 | 5 | 12 | 7 | 14 | -0.255 | 0.799 | -1.173 | 0.241 |
| 54 | 3 | 7 | 8 | 11 | -0.627 | 0.53 | 0.271 | 0.787 |
| 55 | 4 | 9 | 17 | 10 | -1.886 | 0.059 | 1.798 | 0.072 |
| 56 | 5 | 11 | 10 | 12 | -0.873 | 0.383 | -0.232 | 0.817 |
|  |  |  |  |  |  |  |  |  |
| 57 | 11 | 11 | 15 | 38 | 1.786 | 0.074 | 0.698 | 0.485 |
| 58 | 9 | 9 | 9 | 16 | 0.907 | 0.364 | 0 | 1 |
| 59 | 6 | 8 | 11 | 7 | -1.01 | 0.312 | 0.744 | 0.457 |
| 60 | 6 | 12 | 12 | 20 | -0.292 | 0.771 | 0 | 1 |
| 61 | 6 | 6 | 3 | 14 | 1.823 | 0.068 | -0.813 | 0.416 |
| 62 | 4 | 11 | 2 | 7 | 0.238 | 0.812 | -2.591 | **0.01** |
| 63 | 9 | 7 | 8 | 15 | 1.313 | 0.189 | 0.228 | 0.82 |
| 64 | 8 | 11 | 13 | 20 | 0.19 | 0.849 | 0.401 | 0.688 |
|  |  |  |  |  |  |  |  |  |
| 65 | 8 | 10 | 11 | 19 | 0.528 | 0.598 | 0.209 | 0.835 |
| 66 | 10 | 7 | 9 | 11 | 0.827 | 0.408 | 0.462 | 0.644 |
| 67 | 8 | 12 | 4 | 16 | 1.363 | 0.173 | -1.814 | 0.07 |
| 68 | 5 | 6 | 9 | 13 | 0.245 | 0.806 | 0.755 | 0.45 |
| 69 | 4 | 10 | 15 | 17 | -1.147 | 0.251 | 1.081 | 0.28 |
| 70 | 5 | 4 | 5 | 2 | -0.63 | 0.529 | 0.354 | 0.723 |
| 71 | 7 | 8 | 8 | 19 | 1.091 | 0.275 | 0 | 1 |
| 72 | 3 | 13 | 6 | 13 | -0.853 | 0.394 | -1.734 | 0.083 |
|  |  |  |  |  |  |  |  |  |
| 73 | 9 | 17 | 16 | 26 | -0.287 | 0.774 | -0.176 | 0.86 |
| 74 | 14 | 8 | 10 | 16 | 1.72 | 0.085 | 0.406 | 0.684 |
| 75 | 14 | 21 | 19 | 29 | 0.038 | 0.97 | -0.315 | 0.753 |
| 76 | 2 | 7 | 12 | 17 | -1.027 | 0.304 | 1.24 | 0.215 |
| 77 | 1 | 16 | 9 | 16 | -2.222 | **0.026** | -1.626 | 0.104 |
| 78 | 4 | 12 | 12 | 24 | -0.595 | 0.552 | 0 | 1 |
| 79 | 8 | 16 | 15 | 13 | -1.451 | 0.147 | -0.196 | 0.845 |
| 80 | 16 | 13 | 8 | 23 | 2.301 | **0.021** | -0.915 | 0.36 |

*Note:* HH: number of hit-hit; HM: number of hit-miss; MH: number of miss-hit; MM: number of miss-miss; z: z-score; p: p-value; IND: independence; NS: non-stationarity
